# 3D Mesoscale Finite Element Modelling of Concrete under Uniaxial Loadings

**DOI:** 10.3390/ma13204585

**Published:** 2020-10-15

**Authors:** Tiago Forti, Gustavo Batistela, Nadia Forti, Nicolas Vianna

**Affiliations:** 1 Simworx R & D, Campinas 13087-727, Brazil; batistela@simworx.com.br; 2 Exact Sciences, Environmental and Technologies Center, Pontifical Catholic University of Campinas (PUC-Campinas), Campinas 13086-099, Brazil; nadia.cazarim@puc-campinas.edu.br (N.F.); nicolas.jv@puccampinas.edu.br (N.V.)

**Keywords:** finite element method, concrete, mesoscale, damage, cohesive fracture

## Abstract

Concrete exhibits a complex mechanical behavior, especially when approaching failure. Its behavior is governed by the interaction of the heterogeneous structures of the material at the first level of observation below the homogeneous continuum, i.e., at the mesoscale. Concrete is assumed to be a three-phase composite of coarse aggregates, mortar, and the interfacial transitional zone (ITZ) between them. Finite element modeling on a mesoscale requires appropriate meshes that discretize the three concrete components. As the weakest link in concrete, ITZ plays an important role. However, meshing ITZ is a challenging issue, due to its very reduced thickness. Representing ITZ with solid elements of such reduced size would produce very expensive finite element meshes. An alternative is to represent ITZ as zero-thickness interface elements. This work adopts interface elements for ITZ. Damage plasticity model is adopted to describe the softening behavior of mortar in compression, while cohesive fractures describe the cracking process. Numerical experiments are presented. First example deals with the estimation of concrete Young’s modulus. Experimental tests were performed to support the numerical test. A second experiment simulates a uniaxial compression test and last experiment simulates a uniaxial tensile test, where results are compared to data from the literature.

## 1. Introduction

Concrete is a very versatile material, applied in different types of constructions around the world. Some advantages of this material are its ability to mold complex shapes, its fire resistance, and resistance to atmospheric conditions. Economic factors also contribute to the wide use of concrete structures. However, its mechanical behavior is complex. One difficulty in modeling concrete structures is the definition of constitutive laws that are able to describe its nonlinear behavior and the process of cracking.

Concrete exhibits a complex mechanical behavior, especially when approaching failure. Its behavior is mainly governed by the interaction of the heterogeneous structures of the material at the first level of observation below the homogeneous continuum, i.e., at the mesoscale at which concrete is assumed to be a three-phase composite composed of coarse aggregates, mortar, and the interfacial transitional zone (ITZ) between them [1].

Macroscopic material models, where concrete is considered as continuous and homogeneous, are able to reproduce aspects of phenomenological behavior, but are not sufficient to establish cause-effect relationships between geometrical and physical properties of the heterogeneous components and its mechanical response. For this reason, with the increasing capacity of computers, mesoscale modelling has been developed in recent decades [1,2,3,4,5,6,7,8,9,10,11,12].

Finite element modeling on a mesoscale requires appropriate meshes that discretize the three concrete components. Zhou et al. [13] investigated the influence of aggregate shape on concrete mechanical properties. Chen et al. [14] studied the influence of both distribution and geometrical shape of aggregates using 2D planar models. As the weakest link in concrete, it has been recognized that ITZ plays an important role in the macroproperties of concrete because of its higher porosity, lower elastic modulus, and lower tensile strength compared with mortar [6]. However, meshing ITZ is a challenging issue, since ITZ is only 10–50 μm thick. Representing ITZ with solid elements of such reduced size would produce very expensive finite element meshes. In the literature, the ITZ structure is represented by either zero-thickness interface elements [1,5] or oversized solid elements [2]. Maleki et al. [15] investigated the influence of ITZ thickness when modelled as solid elements. In this work, zero-thickness interface elements are adopted.

The concrete mechanical behavior is strongly influenced by the initiation and propagation of internal microcracks. The laws for crack initiation and evolution follow the concepts and principles of Fracture Mechanics. A well-recognized fracture approach for concrete is the cohesive crack implemented in the Fictitious Crack model (FCM) proposed by Hillerborg et al. [16]. Zero-thickness interface elements with appropriate constitutive laws formulated in terms of shear and normal stress and the corresponding relative displacements provide the means to extend the FCM into a modern numerical analysis of fracture in a Finite Element context [1].

This work addresses the mechanical behavior of concrete in a 3D mesoscale finite element model. Softening behavior is represented differently in compression and tension. It proposes to combine damage plasticity to account for compressive softening behavior and interface cohesive fracture elements to account for tensile softening and cracking. The simulations are implemented using the public finite element library PZ. Section 2 presents the governing equations and constitutive laws. Numerical experiments are presented in Section 3. First example deals with the estimation of concrete Young’s modulus. Experimental tests were executed to support the numerical test. A second experiment simulates a uniaxial compression test and last experiment simulates a uniaxial tensile test. Results are in good agreement confirming the strategy of combining damage plasticity and cohesive fractures.

## 2. Governing Equations

The mesoscale simulation requires the description of the three components: coarse aggregate, mortar, and the interfacial transitional zone (ITZ). Coarse aggregate is considered as an elastic material for simplicity because its resistance is much higher than that of mortar for the concretes analyzed in this work. Mortar is described as an elastoplastic material where damage controls the softening of the material in compression. In tension, mortar may crack. For that, cohesive fractures are adopted. The ITZ is described as a zero-thickness interface between aggregate and mortar elements. Thus, the mesh consists of 3D solid elements and 2D interfaces between mortar elements and between mortar and aggregate elements.

### 2.1. Elasticity Problem

The elasticity problem is given by the equilibrium equation:

$$\mathrm{div}\left(\sigma \right)+\vec{b}\;=\vec{0},\;\mathrm{in}\;\Omega$$

where σ is the Cauchy stress tensor, $\vec{b}={\left\{b_{x},b_{y},b_{z}\right\}}^{T}$ are body forces, and $\Omega \subset {\mathbb{R}}^{3}$ is a bounded domain with boundary ∂Ω. Each component of $\vec{b}$ is a function in $L^{2}\left(\Omega \right)$, the space of square-integrable functions. The stress tensor is given, in linear elasticity, by the constitutive law $\sigma =C^{e}:\epsilon$ where ϵ is the infinitesimal strain tensor and $C^{e}$ is the standard isotropic elasticity tensor. Boundary conditions can be prescribed displacements (Dirichlet type) or external forces (Neumann type).

### 2.2. Coarse Aggregates

Coarse aggregate is considered as an elastic material for simplicity because its resistance is much higher than that of mortar for the concretes simulated in this work.

### 2.3. Mortar

Mortar is described as an elastoplastic material. The Mohr-Coulomb yielding criterion is adopted. Softening behavior is represented differently in compression and tension. For compression, a damage model is adopted. For tension, cohesive fractures are adopted.

#### 2.3.1. The Mohr-Coulomb Yielding Criterion

Mortar may exhibit some plastic deformation. Even when simulating a uniaxial test, the stress state of mortar elements is not uniaxial. Thus, an appropriate yielding criterion is necessary. The classic Mohr-Coulomb criterion [17] states that plastic yielding begins when, on plane in the body, the shearing stress τ and the normal stress $\sigma_{n}$ reach the critical combination

$$\tau =c-\sigma_{n}\;\tan\phi$$

where c is the cohesion and ϕ is the angle of internal friction of the material. In terms of the principal stresses $\sigma_{1}>\sigma_{2}>\sigma_{3}$ the yield surface is given by

$$\Phi =\left(\sigma_{1}-\sigma_{3}\right)+\left(\sigma_{1}+\sigma_{3}\right)\sin\phi -2\;c\cos\phi \leq 0$$

For the Mohr–Coulomb criterion to fit a given uniaxial tensile strength $f_{t}$ and a given uniaxial compressive strength $f_{c}$, the parameters ϕ and c are chosen as:

$$\phi =\sin^{-1}\left(\frac{f_{c}-f_{t}}{f_{c}+f_{t}}\right)$$

$$c=\left(\frac{f_{c}{\;f}_{t}}{f_{c}-f_{t}}\right)\tan\phi$$

#### 2.3.2. Compressive Damage Plasticity Model

The Mohr-Coulomb model is simulated, in the numerical tests of this work, without hardening. Thus, in a uniaxial test, the compressive stress would remain constant after yielding. Softening is described by means of a damage plasticity model where damage is function of plastic deformations.

Damage can be defined as a collection of permanent microstructural changes concerning material mechanical properties, such as presence (and development) of microcracks and cavities. Not only is the withstanding capacity of the material affected, but also its stiffness. In this context, the elasticity law is given by

$$\sigma =\left(1-D\right)C^{e}:\epsilon^{e}\;$$

where σ is the stress tensor, D is a scalar value in the range from zero (undamaged material) to one (fully damaged material) representing the current damage of the material, $C^{e}$ is the standard isotropic elasticity tensor, $\epsilon^{e}=\epsilon -\epsilon^{p}$ is the elastic strain tensor, and $\epsilon^{p}$ the plastic strain. The term $\left(1-D\right)C^{e}$ is the damaged stiffness of the material.

There are several possibilities for coupling plasticity and damage effects [18]. In this work, the concept of effective stress is adopted. The plastic yield function is no longer written in terms of the Cauchy stress σ, but it is a function of the effective stress:

$$\sigma_{\mathrm{eff}}=\frac{\sigma }{1-D}$$

This approach provides a simple way to separate the damage and plastic processes. Plastic effects, driven by the effective stresses, can be described independently from damage ones and vice versa [18].

In this work, damage is split into tensile $(D_{t}$) and compressive ($D_{c}$) damages according to $\left(1-D\right)=\left(1-D_{t}\right)\left(1-D_{c}\right)$. Damage is adopted as a function of characteristic strains based on plastic deformations. The characteristic strains $\tilde{\epsilon_{t}}$ and $\tilde{\epsilon_{c}}$ are given in terms of principal plastic strains $\epsilon_{1}^{p}>\epsilon_{2}^{p}>\epsilon_{3}^{p}$ as:

$$\begin{array}{c} \tilde{\epsilon_{t}}=r\;\epsilon_{1}^{p}\\ \tilde{\epsilon_{c}}=-\left(1-r\right)\;\epsilon_{3}^{p} \end{array}$$

where

$$r\;\left(\hat{\sigma }\right)=\{\begin{array}{cc} 0 & \mathrm{if}\hat{\sigma }=0\\ \frac{\sum_{i=1}^{3}{\langle \hat{\sigma_{i}}\rangle }_{+}}{\sum_{i=1}^{3}\left|\hat{\sigma_{i}}\right|} & \mathrm{otherwise} \end{array}$$

is a weight factor (0≤r≤1) as proposed by Lee and Fenves [19]. The Macauley bracket is defined as ${\langle \vartheta \rangle }_{+}=\frac{1}{2}\left(\left|\vartheta \right|+\vartheta \right)$, and $\hat{\sigma_{i}}$ are the principal stresses. If all $\hat{\sigma_{i}}$ are positive, then r=1, and if they are all negative, then r=0. The damage functions $D_{t}\left(\tilde{\epsilon_{t}}\right)$ and $D_{c}\left(\tilde{\epsilon_{c}}\right)$ are computed in order to describe the uniaxial stress–strain curves, which are input data to the simulations.

#### 2.3.3. Cohesive Fractures

In the discrete crack modeling, cracks are modeled as displacement discontinuities between elements. The fictitious crack model [16,20] or cohesive zone model describes the fracture process zone as a discrete crack (fictitious) where softening effects are expressed by cohesive zones in the interface of elements. The crack formation is considered as a gradual phenomenon. After reaching a certain tension stress value, crack starts to open. Then, an inelastic process takes place until the crack reaches a critical value and the crack faces are completely separated. The cohesive stress is defined as function of the relative displacement. Due to tensile stresses, the crack opens perpendicular to the interface element (usually called Mode I fracture). The crack aperture is defined as

$$\left[\vec{u}\right]\cdot \vec{n}=\left[{\vec{u}}_{\mathrm{right}}-{\vec{u}}_{\mathrm{left}}\right]\cdot \vec{u}$$

where ${\vec{u}}_{\mathrm{left}}$ and ${\vec{u}}_{\mathrm{right}}$ are the displacement fields of solid elements neighbor to the interface element and $\vec{n}$ is the vector normal to the interface, pointing from left to right neighbor. The cohesive tension stress is a function of the crack aperture. In this work, the mortar cohesive law is defined using Hordijk’s analytical equation [21]:

$$\sigma_{\mathrm{cohesive}}=\{\begin{array}{cc} f_{t}\left(\left(1+{\left(3\frac{w}{w_{\mathrm{lt}}}\right)}^{3}\right)e^{-6.93\frac{w}{w_{\mathrm{lt}}}}-28\frac{w}{w_{\mathrm{lt}}}e^{-6.93}\right) & \mathrm{for}\;w\leq w_{\mathrm{lt}}\\ 0 & \mathrm{for}\;w>w_{\mathrm{lt}} \end{array}$$

where $w=\left[\vec{u}\;\right]\cdot \;\vec{n}$ is the crack aperture, $w_{\mathrm{lt}}=5.136\frac{G_{F}}{f_{\mathrm{tc}}}$, $f_{t}$ is the mortar tension strength, and $G_{F}$ is the apparent fracture energy, corresponding to the amount of energy per unit area required for the complete separation of the two fracture faces. The implementation follows the description in Forti et al. [22].

### 2.4. Interfacial Transition Zone (ITZ)

The interfacial transition zone (ITZ) is the weakest link between the aggregate and the mortar matrix. The properties of the ITZ are not fully understood yet, however it is well accepted that it has large heterogeneity and high porosity and its strength is lower than the mortar [4]. The elastic modulus of ITZ is about 50–70% of the mortar matrix [6] and ITZ is only 10–50 μm thick. Its strength is also very reduced. Zhou and Hao [4] adopts ITZ strength in both compression and tension as 40% of mortar strength. Shuguang and Qingbin [6] adopts tensile strength of ITZ as 2/3 of mortar strength. Huang et al. [7] adopts Young’s modulus and strength of the ITZ as approximately 75% of mortar strength.

In this work, ITZ is simulated as a zero-thickness interface element. The stress response of the interface element is given as $\sigma \cdot \vec{n}\;=\gamma \;\left[{\vec{u}}_{\mathrm{right}}-{\vec{u}}_{\mathrm{left}}\right]$ where $\gamma =E_{\mathrm{ITZ}}/t_{\mathrm{ITZ}}$, $E_{\mathrm{ITZ}}$ is the Young’s modulus of ITZ, and $t_{\mathrm{ITZ}}$ is its thickness.

Two crack models are implemented for ITZ: a cohesive model (as described in Section 2.3.3) and a brittle crack model. The cohesive model is adopted in the simulation of uniaxial tension test and the brittle model is used in the simulation of the uniaxial compression test in Section 3.

### 2.5. Brittle Crack Model

Bitouri et al. [23] identified that the shear behavior of the interface mortar aggregate follows a linear trend as Mohr-Coulomb model. Motivated by their results, the normal stress is limited by a Mohr–Coulomb yield criterion:

$$\tau_{\mathrm{ITZ}}\leq c-\sigma_{n,\mathrm{ITZ}}\;\tan\phi$$

where $\sigma \cdot \vec{n}\;=\gamma \;\left[{\vec{u}}_{\mathrm{right}}-{\vec{u}}_{\mathrm{left}}\right]$, $\sigma_{n,\mathrm{ITZ}}=\vec{n}\cdot \sigma \cdot \vec{n}$ and $\tau_{\mathrm{ITZ}}^{2}={\left|\sigma \cdot \vec{n}\right|}^{2}-{\left|\sigma_{n,\mathrm{ITZ}}\right|}^{2}$ Once the normal stress $\sigma \cdot \vec{n}$ reaches the Mohr-Coulomb condition, the integration point is set as cracked, and the resulting stress in next load steps is zero.

## 3. Numerical Experiments

The simulations were implemented in C++ language using the object-oriented scientific computational environment PZ (http://github.com/labmec/neopz). PZ is a general finite element approximation software, which incorporates a variety of element geometries, variational formulations, and approximation spaces. It contains modules for a broad-classes of technologies such as system resolution, finite element geometric approximation, finite element approximation spaces (e.g., continuous, discontinuous, H(div), and others), and mesh adaptivity. The library allows the implementation of specific finite element formulations, such as those required for this work, namely, damage plasticity model and cohesive fractures.

### 3.1. Estimating the Young’s Modulus of Concrete

The first numerical experiment describes the estimation of the Young’s modulus of concrete. Experimental tests were performed to provide input material data and reference results. The particle size distribution of coarse aggregates is used to construct the finite element mesh.

#### 3.1.1. Experimental Tests

**Figure 1:**
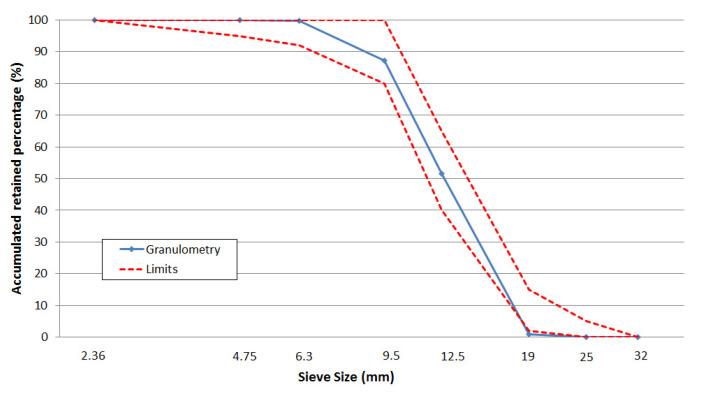
Particle size curve of coarse aggregate.

Materials were characterized by experimental tests. Fine and coarse aggregates were characterized following Brazilian standards. Figure 1 shows the particle size curve of coarse aggregates with their respective limits, as defined by Brazilian Standard NBR NM 248 [24].

The concrete mix is 400 kg/m^3^ of Portland cement (Brazilian type CP V [25]), 809 kg/m^3^ of fine aggregate, 1002 kg/m^3^ of coarse aggregate, and 200 kg/m^3^ of water. A polycarboxylic based superplasticizer was added. Concrete is composed of 57.5% of mortar and 42.5% of coarse aggregate in volume. Mortar samples were also prepared.

Tests indicate an average concrete Young’s modulus of 36,360 MPa and compressive strength of 34.83 MPa. Mortar presented an average Young’s modulus of 28,060 MPa.

#### 3.1.2. Mesh Generation

Mesh generation plays an important role in the mesoscale simulation. Different techniques to construct 2D and 3D mesoscale meshes can be found in the literature [6,7,9,10,11,12,13,26]. We did not have access to tools, such as X-ray computed tomography, which are able to recreate exactly the laboratory specimen in a computational environment. Instead, we opted to create a new concrete mesh for the purpose of this study, using the aggregate-to-cement ratio and the particle size curve from the original test mix.

**Figure 2:**
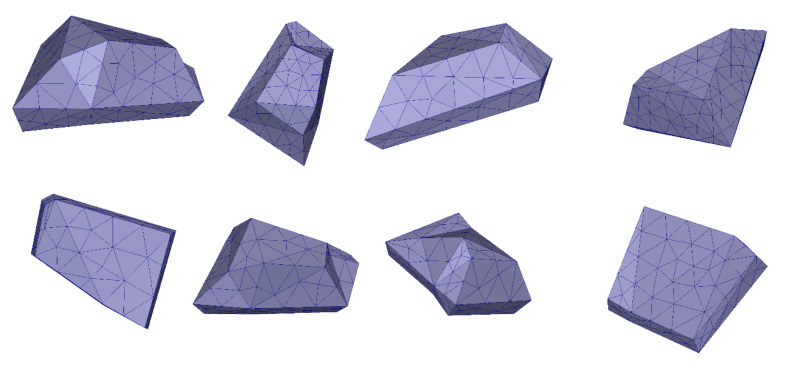
Example of randomly generated aggregate shapes.

The first step was to develop an aggregate shape generator, to create a variety of polyhedrons by randomly tweaking geometric parameters such as the number of vertices and their coordinates. This was an empirical stage, and the parameters range were set based on the visual resemblance between the resulting shapes and actual aggregates. The product of this step is shown in Figure 2, which depicts 8 example aggregates.

Fifty shapes were generated and scaled accordingly to match the actual concrete mix. The reference concrete was the one used in the laboratory tests. From the test mix, we obtained the aggregate-to-cement ratio and the particle size curve (Figure 1).

**Figure 3:**
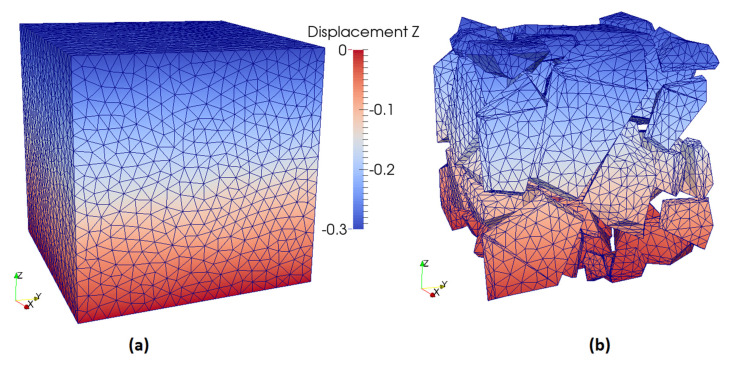
Case #1 simulation. Single block mesh. (**a**) mesh view; (**b**) view of aggregate elements.

A cube of side length of 46.4 mm was created, with the dimensions required to accommodate the 50 aggregates with the restriction of the aggregate-to-cement ratio. To determine the scaling factor of the aggregates, their minimum dimension was compared to the nominal aperture size of the desired sieve. For every sieve of the granulometric curve, new shapes were created until their correspondent aggregate volume was reached. After all the shapes were created, they were manually inserted inside the cube specimen and checked for noncolliding objects. It created the single block mesh, as shown in Figure 3.

**Figure 4:**
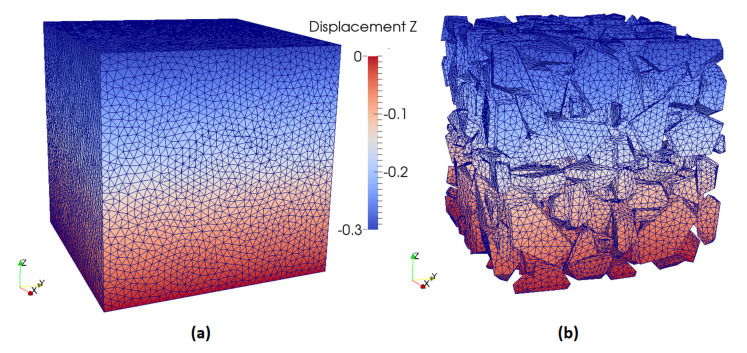
Case #4 simulation; 8-blocks mesh. (**a**) mesh view; (**b**) view of aggregate elements

The single block mesh is used to construct a larger mesh. The set of aggregates was multiplied (8 blocks) and disposed into a cube twice the original side length, i.e., 92.8 mm, that ended up with 400 aggregates objects. At every insertion, the set of aggregates was rotated in a different way to eliminate symmetry as much as possible. The positions of the aggregates that were originally close to the boundary were tweaked once they were inserted into the bigger cube. This was necessary to avoid having sections planes with no aggregate in middle of the bigger cube. The obtained 8-blocks mesh is shown in Figure 4. The FEM mesh, which is composed of tetrahedra elements and triangle elements for boundary condition, was created using the software Gmsh [27]. Gmsh is a free, open-source program capable of generating meshes of different geometry and with various advanced options to enhance element quality.

#### 3.1.3. Simulations

In order to estimate the Young’s modulus of concrete, it is not necessary to consider all the non-linear features described in Section 2. Thus, a linear elastic simulation takes place. Coarse aggregate is simulated with Young’s modulus between $E_{\mathrm{agg}}=50$ and $E_{\mathrm{agg}}=60\;\mathrm{GPa}$ and Poisson’s ratio of 0.17, which are similar to values adopted by Shuguang and Qingbin [6], Huang et al. [7], Grote et al. [28], and Contrafatto et al. [8]. Mortar has a Young’s modulus of 28,060 MPa, according to experimental tests, and Poisson’s ratio of 0.2. The ITZ is simulated as a zero-thickness interface. The stress response of the interface element is given as $\sigma \cdot \vec{n}=\gamma \;\left[{\vec{u}}_{\mathrm{right}}-{\vec{u}}_{\mathrm{left}}\right]$ where $\gamma =E_{\mathrm{ITZ}}/t_{\mathrm{ITZ}}$ $E_{\mathrm{ITZ}}$ is the Young’s modulus of ITZ, and $t_{\mathrm{ITZ}}$ is its thickness.

First simulations were carried out with $E_{\mathrm{ITZ}}=18706.7\;\mathrm{MPa}$, which is 2/3 of mortar’s, $t_{\mathrm{ITZ}}=50\;\mu m$ and $E_{\mathrm{agg}}=60\;\mathrm{GPa}$. Table 1 compares the obtained Young’s modulus of concrete $E_{\mathrm{concrete}}$ for different finite element meshes. It aims to assess the quality of solution approximations. Cases #1, #2, and #3 simulated the single block mesh. Case #1 adopted approximation order p=1. Case #2 improves the approximation order to p=2. Case #3 uses p=1 and refine all mesh elements. Case #4 solves the 8-blocks mesh, with p=1. It can be noted that the resulting $E_{\mathrm{concrete}}$ are very similar among them. It can be considered that mesh used in simulation case #1 is adequate to simulate the linear elastic behavior of the problem and calculate $E_{\mathrm{concrete}}$. Thus, mesh of case #1 is adopted to next simulations.

Table 2 compares the obtained $E_{\mathrm{concrete}}$ for different $E_{\mathrm{ITZ}}$ and $t_{\mathrm{ITZ}}$, with $E_{\mathrm{agg}}=60\;\mathrm{GPa}$. As expected, the higher $E_{\mathrm{ITZ}}$, the greater the obtained $E_{\mathrm{concrete}}$. Moreover, the thinner ITZ, the higher the $E_{\mathrm{concrete}}$. However, the dispersion of results is very small, the maximum value obtained for $E_{\mathrm{concrete}}$ being only 2% higher than the lowest. In addition, the numerical results are in good agreement with experimental tests where the average modulus is $E_{\mathrm{concrete}}^{\mathrm{experimental}}=36,360\;\mathrm{MPa}$.

Table 3 compares the obtained $E_{\mathrm{concrete}}$ for different $E_{\mathrm{ITZ}}$ and $t_{\mathrm{ITZ}}$, with $E_{\mathrm{agg}}=50\;\mathrm{GPa}$. The results of concrete Young’s modulus are slightly lower than experimental $E_{\mathrm{concrete}}^{\mathrm{experimental}}=36,360\;\mathrm{MPa}$.

Thus, predicted values are between 35,401 and 39,028 MPa while experimental testes indicate an average concrete Young’s modulus of 36,360 MPa.

### 3.2. Modelling of Uniaxial Compression Test

The mesoscale model is applied to a uniaxial compression test and compared to the simulations of Huang et al. [7]. The simulations are performed using the finite element mesh described in Section 3.1.2. The mesh is not the same of Huang et al. [7]. The particle size distribution is different and their concrete is about 46% of aggregates, whereas in this work, we have about 42% of aggregates. However, adopting same material data, it is possible to compare results. No voids or initial cracks, which may exist in concrete, were considered in the simulations of this work.

#### 3.2.1. Material Data

In this 3D mesoscale model, concrete is assumed to be a three-phase composite composed of coarse aggregates, mortar, and the interfacial transitional zone (ITZ) between them. The adopted data for each phase is described, based on Huang et al. [7].

Coarse aggregate is considered as an elastic material. The elastic parameters are adopted with Young’s modulus of 50 GPa and Poisson’s ratio of 0.17.

**Figure 5:**
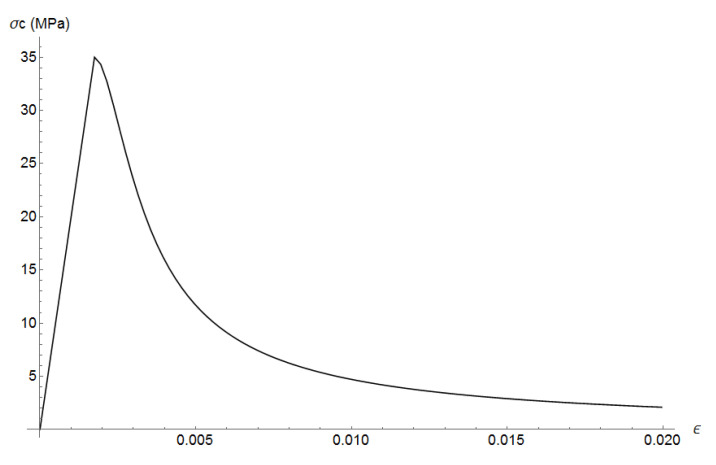
Mortar: uniaxial compressive stress-strain relation.

**Figure 6:**
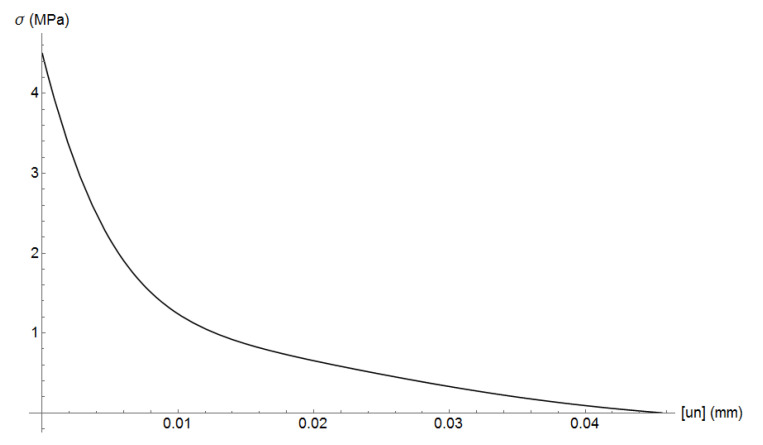
Mortar cohesive curve: tension stress versus crack aperture.

Mortar is described as an elastoplastic material. The nonassociative Mohr-Coulomb yielding criterion is adopted with cohesion c=6.27 MPa, angle of internal friction ϕ=50.55°, and material dilatation angle of 31°. These cohesion and internal friction angle fit the given uniaxial tensile strength $f_{t}=4.5\;\mathrm{MPa}$ and uniaxial compressive strength $f_{c}=35\;\mathrm{MPa}$. Softening behavior is represented differently in compression and tension. For compression, a damage model is adopted, and for tension, cohesive fractures are adopted. The prepeak compressive stress–strain curve is assumed linear elastic for simplicity and the following equation [7] describes the postpeak softening curve:

$$\sigma_{c}=f_{c}\;\left(\frac{\frac{\epsilon }{\epsilon_{c}}}{\alpha {\left(\frac{\epsilon }{\epsilon_{c}}-1\right)}^{2}+\frac{\epsilon }{\epsilon_{c}}}\right)$$

where $\sigma_{c}$ and ϵ are the compressive stress and strain, $f_{c}=35\;\mathrm{MPa}$ is the compressive strength, $\epsilon_{c}=f_{c}/E$ is the compressive strain, E=20 GPa is mortar’s Young’s modulus, and $\alpha =0.157{\;f}_{c}^{0.785}-0.905$. Figure 5 plots the uniaxial compressive stress-strain relation. Mortar’s tensile strength is assumed as $f_{t}=4.5\;\mathrm{MPa}$. The cohesive curve adopts Hordijk’s curve with fracture energy of $G_{F}=40\;J/m^{2}$. The cohesive tension stress curve is plotted in Figure 6.

The interfacial transition zone (ITZ) is simulated as a zero-thickness interface element with a brittle crack model. The stress response of the interface element is given as $\sigma \cdot \vec{n}=\gamma \;\left[{\vec{u}}_{\mathrm{right}}-{\vec{u}}_{\mathrm{left}}\right]$ where $\gamma =E_{\mathrm{ITZ}}/t_{\mathrm{ITZ}}$, $E_{\mathrm{ITZ}}=15\;\mathrm{GPa}$ is the Young’s modulus of ITZ, and $t_{\mathrm{ITZ}}=50\;\mu m$ is its thickness. ITZ’s uniaxial compressive and tensile strengths are adopted as 27 and 3.5 MPa. Thus, the Mohr–Coulomb parameters cohesion c and the angle of internal friction ϕ are c=4.86 MPa and ϕ=50.40°.

#### 3.2.2. Results

**Figure 7:**
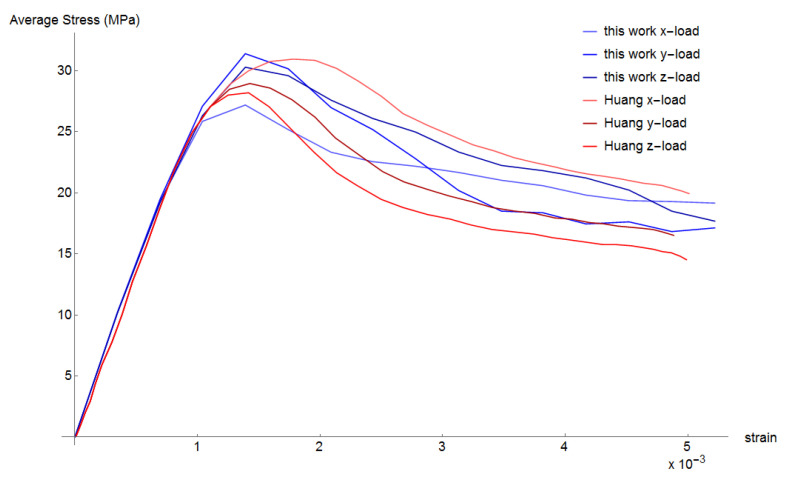
Simulated stress–strain curves for uniaxial comparison.

**Figure 8:**
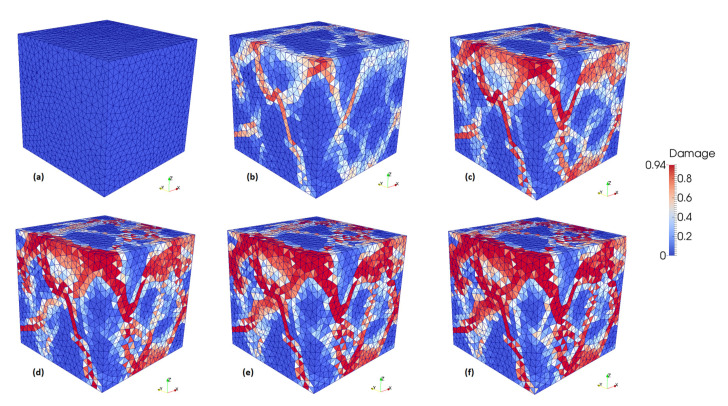
Compression damage evolution (from **a** to **f**) for load applied in z-direction.

Vertical displacement is applied to all nodes on the upper face of the mesh and the opposite face is fixed vertically. The reaction force divided by the cross-section area provides an equivalent stress and stress-strain curves are obtained. The vertical load was applied in the three axes directions (x, y, and z). The predicted strength values are 27.2, 30.2, and 30.3 MPa, respectively. Huang et al. [7] also loaded in the three axes directions obtaining strength values of 31.0, 29.0, and 28.2 MPa. Figure 7 compares the obtained curves to the simulated curves of Huang et al. [7]. It can be observed that results are in good agreement. The compression damage evolution for the case when load was applied in z-direction is shown in Figure 8.

### 3.3. Tensile Test

The 3D mesoscale model is applied to a uniaxial tension test. Input data is the same as the previous compression test (Section 3.2), except from ITZ model. Here, ITZ is modelled as cohesive fractures. Results are compared to those of Huang et al. [7].

**Figure 9:**
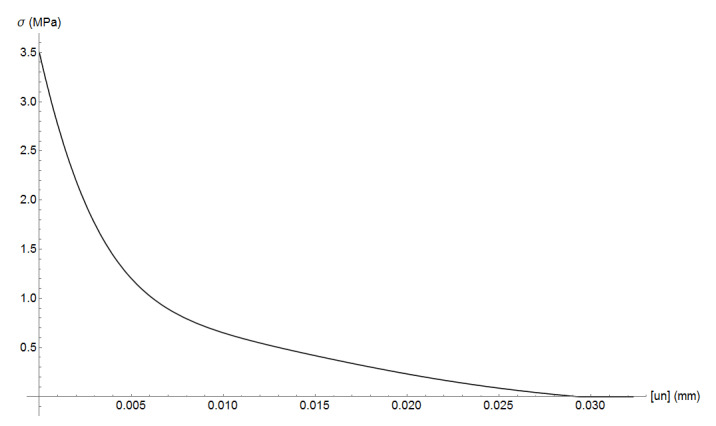
Interfacial transitional zone (ITZ) cohesive curve: tension stress versus crack aperture.

**Figure 10:**
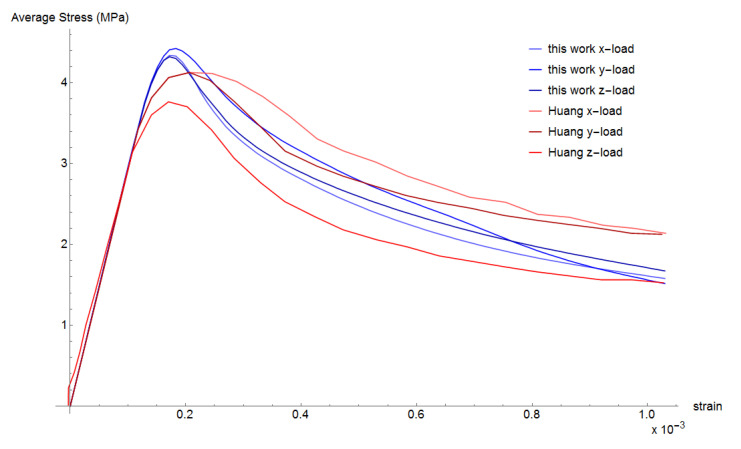
Simulated stress–strain curves for uniaxial tension test.

**Figure 11:**
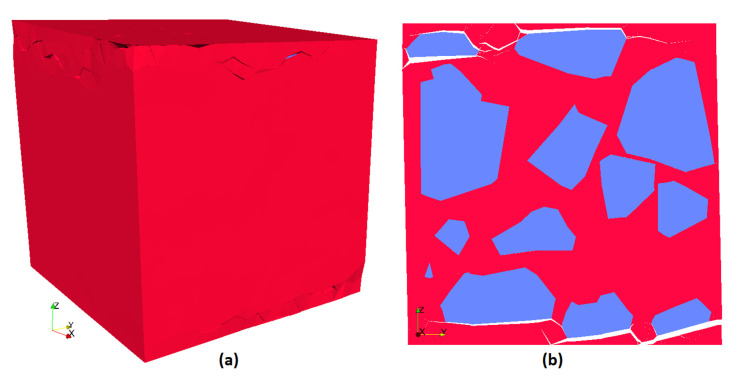
Final deformed shape for load applied in z-direction: (**a**) 3D view and (**b**) slice view.

ITZ is modelled as a cohesive fracture following Hordijk [21] equation with tensile strength $f_{t}=3.5\;\mathrm{MPa}$ and fracture energy of $G_{F}=20\;J/m^{2}$, according to Huang et al. [7]. The cohesive tension stress curve is plotted in Figure 9 and Figure 10 plots the obtained stress-strain curves and those of Huang et al. [7]. It can be observed that results are similar and in good agreement. The predicted tensile strength values are 4.34, 4.42, and 4.32 MPa, which are slightly higher than the predicted values of Huang et al. [7] that are 3.78, 4.15, and 4.13 MPa. In the softening part, the obtained curves are also similar to that of Huang et al. [7]. The deformed shape for the case when load was applied in z-direction is plotted in Figure 11. It can be observed that cracks start in ITZ interface and then propagate through mortar elements.

## 4. Conclusions

A 3D mesoscale finite element model of concrete was developed. Concrete is assumed to be a three-phase composite composed of coarse aggregates, mortar, and the interfacial transitional zone (ITZ) between them. The constitutive laws for each of the three phases are described. A damage plasticity model is adopted to describe the compressive behavior of mortar. In tension, mortar softening is described by a cohesive fracture model. Coarse aggregates are modelled as a linear elastic material, for simplicity, but the nonlinear framework developed for mortar could be applied to coarse aggregates. Special attention is devoted to ITZ. ITZ is the weakest link between the aggregate and the mortar matrix. The properties of the ITZ are not fully understood yet, however, it is well accepted that it has large heterogeneity, high porosity, and its strength is lower than the mortar. Modeling ITZ as 3D solid elements is a challenging issue, since ITZ is only 10–50 μm thick, what would produce very expensive finite element meshes. An alternative is to represent ITZ by zero-thickness interface elements, which is adopted in this work. The ITZ thickness and Young’s modulus are taken into account when defining the interface constitutive law. Two crack models are implemented for ITZ: a cohesive model (similar to mortar’s) and a brittle crack model.

Numerical experiments are presented. First example deals with the estimation of concrete Young’s modulus. Experimental tests were executed to support the numerical test. The mesh generation process is described. The coarse aggregate size distribution is represented in the finite element mesh. Simulations were carried out varying ITZ properties and mesh refinement. Predicted values are between 35,401 and 36,033 MPa, when aggregates are simulated with Young’s modulus of $E_{\mathrm{agg}}=50\;\mathrm{GPa}$, and between 38,228 and 39,028 MPa for $E_{\mathrm{agg}}=60\;\mathrm{GPa}$. These results are in good agreement with the experimental tests, which indicate an average concrete Young’s modulus of 36,360 MPa.

A second experiment simulates a uniaxial compression test from Huang et al. [7]. The predicted strength values are 27.2, 30.2, and 30.3 MPa, which are similar to Huang et al.’s [7] obtained strength values of 31.0, 29.0, and 28.2 MPa. The obtained stress-strain curve is compared to the simulated curves of Huang et al. [7], from which it is possible to observe that the softening part of the uniaxial stress-strain curves is also in good agreement.

Last experiment simulates a uniaxial tensile test, also from Huang et al. [7]. The predicted tensile strength values are 4.34, 4.42, and 4.32 MPa, which are slightly higher than the predictions of Huang et al. [7] that are 3.78, 4.15, and 4.13 MPa. In the softening part, the obtained stress-strain curves are also similar.

The results confirm the numerical strategies and models proposed. The use of interface elements for ITZ led to good results in simulating both the Young’s modulus problem and uniaxial strength tests. Results also confirm the strategy of combining compressive damage plasticity and cohesive fractures.

## Figures and Tables

**Table 1:** Calculated Young’s modulus of concrete. $E_{\mathrm{ITZ}}=2/3{\;E}_{\mathrm{mortar}}$, $t_{\mathrm{ITZ}}=50\;\mu m$, and $E_{\mathrm{agg}}=60\;\mathrm{GPa}$. Experimental value is 36,360 MPa.

Case ID	Mesh Parameters	Number of Equations	Number of Coarse Aggregate Elements	Number of Mortar Elements	Number of ITZ Elements	$E_{\mathrm{concrete}}(\mathrm{MPa})$
#1	Single block, p=1	64,980	33,299	38,758	15,888	38,662
#2	Single block, p=2	400,398	33,299	38,758	15,888	38,285
#3	Single block, p=1, refined mesh	400,398	199,794	232,548	63,552	38,392
#4	8-blocks, p=1	386,883	203,986	224,321	101,186	38,810

**Table 2:** Calculated Young’s modulus of concrete with different ITZ data. $E_{\mathrm{agg}}=60\;\mathrm{GPa}$. Mesh: single block, p=1. Experimental value is 36,360 MPa.

Case ID	$E_{ITZ}$	$t_{ITZ}\;$ (μm)	Young’s Modulus of Concrete (MPa)
#1	$2/3E_{\mathrm{mortar}}$	50	38,662
#5	$1/2E_{\mathrm{mortar}}$	50	38,515
#6	$1/3E_{\mathrm{mortar}}$	50	38,228
#7	$2/3E_{\mathrm{mortar}}$	10	39,028
#8	$1/2E_{\mathrm{mortar}}$	10	38,997
#9	$1/3E_{\mathrm{mortar}}$	10	38,935

**Table 3:** Calculated Young’s modulus of concrete with different ITZ data. $E_{\mathrm{agg}}=50\;\mathrm{GPa}$. Mesh: single block, p=1. Experimental value is 36,360 MPa.

Case ID	$E_{ITZ}$	$t_{ITZ}\;$ (μm)	Young’s Modulus of Concrete (MPa)
#10	$2/3E_{\mathrm{mortar}}$	50	35,745
#11	$1/2E_{\mathrm{mortar}}$	50	35,628
#12	$1/3E_{\mathrm{mortar}}$	50	35,401
#13	$2/3E_{\mathrm{mortar}}$	10	36,033
#14	$1/2E_{\mathrm{mortar}}$	10	36,008
#15	$1/3E_{\mathrm{mortar}}$	10	35,960
